# Correction: Teeuwssen and Fodde; Colon Cancer Heterogeneity and Phenotypic Plasticity in Metastasis Formation. *Cancers* 2019, *11*(9), 1368

**DOI:** 10.3390/cancers12061392

**Published:** 2020-05-28

**Authors:** Miriam Teeuwssen, Riccardo Fodde

**Affiliations:** Department of Pathology, Erasmus MC Cancer Institute, Erasmus University Medical Center, 3015 GD Rotterdam, The Netherlands; m.teeuwssen@erasmusmc.nl

The authors would like to make a correction to their published paper [1].

The authors would like to change one incorrect sentence in reference [1].

On page 9, in paragraph 2, the sentence “In colon cancer, CAFs release exosomes containing miR-92a-3p and promote invasion and chemotherapy resistance. miR-92a-3p directly binds to FBXW7 and MOAP1 thereby activating Wnt-induced EMT and mitochondrial apoptosis [89].” should be changed to “In colon cancer, CAFs release exosomes containing miR-92a-3p and promote invasion and chemotherapy resistance. miR-92a-3p directly binds to FBXW7 and MOAP1 thereby activating Wnt-induced EMT and inhibiting mitochondrial apoptosis [89].”

The change does not affect the scientific results.

The rest of the manuscript does not to be changed. The authors would like to apologize for any inconvenience caused. The manuscript will be updated, and the original will remain available on the article webpage.
